# Two Multidrug-Resistant *Escherichia coli* Isolates from Blood Cultures After Cervical Conization in a Patient with Cervical Intraepithelial Neoplasia Grade II: Phenotypic and Genomic Characterization

**DOI:** 10.3390/pathogens15050476

**Published:** 2026-04-28

**Authors:** Jingrui Zhang, Xiao Liu, Li Liu, Yeshun Fan, Zhiwen Sun, Mengjie Li, Yanwen Xiong, Zengbin Liu, Yanfang Li, Lili Yu, Hongru Feng, Duochun Wang, Jingxiao Zhang

**Affiliations:** 1 Department of Clinical Laboratory, The Fourth Hospital of Shijiazhuang, Shijiazhuang 050011, China; jingrui20005@163.com (J.Z.); liuliok2012@163.com (L.L.); 2 National Key Laboratory of Intelligent Tracking and Forecasting for Infectious Diseases, National Institute for Communicable Disease Control and Prevention, Chinese Center for Disease Control and Prevention, Beijing 102206, China; liuxiao930919@163.com (X.L.); fanyeshun2000@163.com (Y.F.); sunzhiwen@icdc.cn (Z.S.); lmengjie2023@lzu.edu.cn (M.L.); xiongyanwen@icdc.cn (Y.X.); 3 School of Public Health, Lanzhou University, Lanzhou 730000, China; 4 Department of Clinical Laboratory, The Second Hospital of Hebei Medical University, Shijiazhuang 050061, China; liuzengbin0727@163.com; 5 Department of Gynecology, The Second Hospital of Hebei Medical University, Shijiazhuang 050061, China; yf_lee1987@126.com; 6 Department of Neurology, The Second Hospital of Hebei Medical University, Shijiazhuang 050061, China; 28704968@hebmu.edu.cn; 7 Department of The North Campus, The Second Hospital of Hebei Medical University, Shijiazhuang 050061, China; fenghongru2024@163.com; 8 Department of Gynecology, The Fourth Hospital of Shijiazhuang, Shijiazhuang 050011, China

**Keywords:** multidrug-resistant *Escherichia coli*, *bla*_NDM-5_, comparative genomics, blood cultures, carbapenem resistance

## Abstract

Patients undergoing cervical conization for cervical intraepithelial neoplasia grade II (CIN II) may develop postoperative bloodstream infections (BSIs), and multidrug-resistant (MDR) *Escherichia coli* isolates with distinct resistance profiles can complicate antimicrobial management. In this case-based study, two *E. coli* strains, HBFY-1 and HBFY-2, were recovered from blood cultures obtained from a 38-year-old CIN II patient with postoperative fever. The isolates were characterized by antimicrobial susceptibility testing, whole-genome sequencing, conjugation assays, and comparative genomics against publicly available genomes matched by sequence type and serotype. Fever occurred on postoperative day 2. HBFY-1 was fluoroquinolone-resistant; belonged to ST744/O101:H9; carried the extended-spectrum β-lactamase (ESBL) gene *bla*_CTX-M-27_; was phenotypically confirmed as an ESBL producer; and grouped within a multi-source near-neighbor clade consistent with a conserved fluoroquinolone-associated resistance backbone in ST744/O101:H9. HBFY-2 was carbapenem-resistant; belonged to ST48/O113:H32; carried *bla*_NDM-5_ on an IncY-associated plasmid bin; was phenotypically confirmed as a metallo-carbapenemase producer; and did not harbor any ESBL gene. Within the matched ST48/O113:H32 dataset, *bla*_NDM-5_ was detected only in HBFY-2, which clustered within an Asia-enriched lineage, including China-derived human and swine genomes. The *bla*_CTX-M-27_-associated and *bla*_NDM-5_-associated elements were transferred to *E. coli* C600 at frequencies of 5.3 × 10^−2^ and 4.6 × 10^−6^, respectively, and transfer of the *bla*_NDM-5_-associated element imposed no detectable growth penalty under the tested conditions. As this study is based on a single clinical case, the findings should be interpreted cautiously, yet they still highlight the potential value of integrating susceptibility testing with rapid genomic characterization for identifying mobilizable carbapenem-resistance platforms.

## 1. Introduction

Cervical intraepithelial neoplasia (CIN) is a premalignant lesion driven by persistent infection with high-risk human papillomavirus (HPV) and is a recognized precursor to cervical cancer [1,2]. High-grade CIN, including cervical intraepithelial neoplasia grade II (CIN II), is commonly managed by excisional procedures such as cervical conization [3,4]. Although generally safe, excisional treatment can disrupt the cervical mucosal barrier and, together with perioperative factors, may transiently increase susceptibility to postoperative infectious complications, including bacteremia caused by opportunistic *Enterobacterales* [5,6].

Bloodstream infections (BSIs) are serious invasive infections associated with substantial clinical burden [7]. In recent decades, management has been further complicated by the emergence and international spread of multidrug-resistant (MDR) Gram-negative pathogens, including carbapenemase-producing *Escherichia coli* [8,9]. In *E. coli*, resistance to expanded-spectrum cephalosporins is most commonly mediated by extended-spectrum β-lactamases (ESBLs), particularly CTX-M-type enzymes, whereas carbapenem resistance is frequently associated with carbapenemase production, including mobile *bla*_NDM_ determinants [10,11,12]. Fluoroquinolone resistance typically arises through chromosomal mutations in the quinolone resistance-determining regions (QRDRs) of *gyrA* and *parC* and may be further augmented by plasmid-mediated quinolone resistance determinants such as *qnr* genes and *aac(6′)-Ib-cr* [13,14]. Among carbapenemases, New Delhi metallo-β-lactamase (NDM) has garnered particular attention due to its capacity to compromise last-line treatment options [15]. Variants such as *bla*_NDM-5_ are frequently carried on mobile plasmids, facilitating horizontal transfer across *E. coli* lineages. IncX3 plasmids have been repeatedly implicated in the dissemination of *bla*_NDM-5_, and IncF-type backbones have also been reported in certain settings [16,17,18]. In China, *bla*_NDM-5_ has been reported in *E. coli* from animal-associated environments, including poultry and waterfowl farms, and ST48 has also been described in such reservoirs [19,20]. However, the transferability, stability, and potential fitness cost of *bla*_NDM-5_-associated elements in specific genetic backgrounds, including ST48, remain incompletely defined.

From a population-genomic perspective, ST48 and ST744 represent two *E. coli* lineages that have been reported across different settings and may follow distinct resistance trajectories. ST48 has been described in healthcare-associated infections and can acquire multiple resistance determinants [21,22], whereas ST744 has been detected in diverse sources, including human and non-human collections, and has also been reported among bloodstream isolates [23,24]. The recovery of two distinct *E. coli* isolates from blood cultures collected during the postoperative episode allowed a case-based comparison of lineage-associated resistance features under a shared clinical context. However, this case-based observation does not permit definitive confirmation of mixed bloodstream infection and should be interpreted within the limits of the available evidence.

In this study, conducted at the Fourth Hospital of Shijiazhuang, Hebei Province, we describe a case of postoperative fever after cervical conization in a patient with CIN II, from whose blood cultures two *E. coli* isolates were recovered on 30 September 2024. This case-based study combined clinical description with detailed phenotypic and genomic characterization of the two clinical isolates. Antimicrobial susceptibility testing, whole-genome sequencing, conjugation assays, and fitness assays were used to characterize the two isolates and to place them in a phylogenomic context using publicly available genomes matched by ST and O:H serotype. Given the case-based design, our interpretation was restricted to isolate-level phenotypic and genomic characterization and was not intended to establish a definitive mixed bloodstream infection.

## 2. Materials and Methods

### 2.1. Clinical Indicators, Clinical Samples, Bacterial Isolates, and Identification

Blood samples were collected from a 38-year-old female diagnosed with cervical high-grade squamous intraepithelial lesion (HSIL; CIN II) complicated by human papillomavirus type 16 (HPV-16) infection. The patient developed a fever (38.3 °C) on postoperative day 2 following cervical conization. Hematological parameters were measured by the Sysmex XN-10 Automated Hematology Analyzer (Sysmex, Shanghai, China). C-reactive protein (CRP) was measured using a Lifotronic PA-900 specific protein analyzer (Lifotronic, Shenzhen, China). Procalcitonin was quantified using the Pylon3D cyclic enhanced fluorescence immunoassay system (ET Healthcare, Suzhou, China). Blood cultures were performed using the bioMérieux BACT/ALERT 3D120 microbial detection system (bioMérieux, Marcy-l’Étoile, France). Blood samples from positive blood culture bottles were inoculated onto Columbia blood agar (Babio, Jinan, China), China Blue agar (Babio, Jinan, China), and chocolate agar plates (Babio, Jinan, China) and incubated aerobically at 37 °C for 18–24 h. Two *E. coli* isolates (HBFY-1 and HBFY-2) obtained from these blood cultures were identified using the VITEK 2 Compact system (bioMérieux, Marcy-l’Étoile, France) with GN identification cards and further confirmed by matrix-assisted laser desorption ionization time-of-flight mass spectrometry (MALDI-TOF MS) and 16S rDNA sequencing. Sequence analysis was performed using the Basic Local Alignment Search Tool (BLAST+ 2.17.0) [25].

### 2.2. Antimicrobial Susceptibility Testing (AST) and Phenotypic Enzyme Detection

VITEK 2 Compact GN-09 and N335 cards (bioMérieux) were used to determine the minimum inhibitory concentrations (MICs) of the bacteria. MICs for colistin, cefiderocol, ceftazidime–avibactam, and imipenem–relebactam were assessed using the broth microdilution method. A total of 24 antibiotics were tested, including ampicillin, piperacillin, ceftazidime–avibactam, imipenem–relebactam, ampicillin/sulbactam, ticarcillin/clavulanic acid, piperacillin/tazobactam, cefoperazone/sulbactam, aztreonam, cefiderocol, cefazolin, cefepime, cefotetan, cefuroxime, ceftazidime, ceftriaxone, amikacin, gentamicin, tobramycin, ciprofloxacin, levofloxacin, colistin, imipenem, and meropenem. The antimicrobial susceptibilities were interpreted according to the standards of the Clinical and Laboratory Standards Institute (CLSI, 2025). *E. coli* ATCC 25922 and *Pseudomonas aeruginosa* ATCC 27853 were used as the quality control strains. Phenotypic confirmation of ESBL production was performed using the combined disk test (CDT) [26,27]. Carbapenemase production was assessed using the modified carbapenem inactivation method (mCIM) and the EDTA-modified carbapenem inactivation method (eCIM), according to CLSI guidelines [28,29,30].

### 2.3. Conjugation Assay and Growth Curve Analysis

Conjugation experiments were conducted to assess the transferability of *bla*_NDM-5_-carrying element from *E. coli* HBFY-2 and the *bla*_CTX-M-27_-carrying element from HBFY-1 using filter mating [31]. *E. coli* C600, kindly provided by Professor Xiaoping Liao (South China Agricultural University), was used as the recipient strain. For HBFY-2, transconjugants were selected on MacConkey agar containing streptomycin (1500 µg/mL) and meropenem (2 µg/mL) and were confirmed as *E. coli* by MALDI-TOF MS and as *bla*_NDM-5_-positive by PCR. For HBFY-1, transconjugants were selected on MacConkey agar containing streptomycin (1500 µg/mL) and cefotaxime (2 µg/mL) and were confirmed as *E. coli* by MALDI-TOF MS and as *bla*_CTX-M-27_-positive by PCR. Antimicrobial susceptibility testing of the recipient strain and transconjugants was performed as described above. The conjugation frequency (CF) was calculated as follows: CF = (number of transconjugant bacterial colonies × 10x)/(number of recipient bacterial colonies × 10y), where x and y represent the dilution factors.

Growth characteristics were assessed by growth curve analysis. Growth kinetics of *E. coli* strains HBFY-1, HBFY-2, C600, and the HBFY-2 transconjugant (HBFY-2T) were measured in LB broth over 24 h using a Bioscreen C Automated Growth Curve Analysis System (FP-1100-C, Oy Growth Curves Ab Ltd., Helsinki, Finland) at 37 °C by monitoring OD_600_. All experiments were performed in triplicate. Data are presented as mean ± standard deviation (SD). Growth curves were used to compare the growth performance of HBFY-1 and HBFY-2 and to evaluate the potential fitness cost associated with the transfer of the *bla*_NDM-5_-carrying element. Growth differences were analyzed by one-way analysis of variance (ANOVA) followed by Tukey’s multiple-comparison test. A two-sided *p*-value < 0.05 was considered statistically significant.

### 2.4. Whole-Genome Sequencing (WGS), Molecular Characterization, and Bioinformatics Analysis

Genomic DNA was extracted using the Wizard^®^ Genomic DNA Purification Kit (Promega, Madison, WI, USA). DNA concentration was measured using a Qubit fluorometer (Thermo Fisher Scientific, Waltham, MA, USA). Sequencing libraries were prepared using the NEBNext^®^ Ultra™ II DNA Library Prep Kit for Illumina^®^ (New England Biolabs, Ipswich, MA, USA), following the manufacturer’s instructions. Whole-genome sequencing was performed on an Illumina NovaSeq 6000 platform with 150 bp paired-end reads (PE150).

Raw reads were quality-controlled using fastp v1.0.1, with poly-G trimming enabled. Read pairs were discarded if either read contained more than 15 ambiguous bases, more than 50% low-quality bases (Q ≤ 5), or detectable adapter contamination. Reads shorter than 150 bp after filtering were discarded. De novo assembly was performed using SPAdes v3.13.1 with k-mer sizes of 33, 45, 55, and 65 [32]. The optimal assembly result was selected based on assembly continuity and completeness. Assembly quality was assessed using QUAST v5.2.0 [33]. Bacterial strain identities were confirmed through average nucleotide identity (ANI) calculations and digital DNA-DNA hybridization (dDDH) for genetic relationships [34,35]. Annotation was performed using Prokka v1.14.6 [36]. The multilocus sequence type (MLST) and serotype were assigned using PubMLST (https://pubmlst.org/organisms/escherichia-spp; accessed on 5 February 2026) and SerotypeFinder v2.0 [37,38]. Acquired antimicrobial resistance genes were identified using AMRFinderPlus v4.2.7 with default thresholds [39]. The presence of *bla*_NDM-5_ in HBFY-2 was further confirmed by PCR amplification and Sanger sequencing using previously described primers [40]. Plasmid contigs were reconstructed and binned using MOB-suite v3.1.9 [41].

### 2.5. Phylogenomic Analysis of Global E. coli ST744/O101:H9 and ST48/O113:H32

Publicly available *E. coli* genome assemblies and metadata were downloaded from EnteroBase (https://enterobase.warwick.ac.uk; accessed on 30 January 2026) by querying ST744 and ST48 [42]. Assemblies were then filtered by serotype prediction using SerotypeFinder v2.0, retaining ST744/O101:H9 and ST48/O113:H32 genomes for downstream analyses.

Core-genome SNPs were called with Snippy v4.6.0 (https://github.com/tseemann/snippy; accessed on 10 February 2026), with the corresponding clinical isolate as the reference genome, followed by recombination filtering with Gubbins v3.4.3 [43]. Maximum-likelihood phylogenies were inferred using IQ-TREE v2.4.0 with 1000 bootstrap replicates and visualized in iTOL [44,45]. For ST744/O101:H9, a recombination-filtered cgSNP distance threshold of ≤60 from HBFY-1 was used as an operational criterion to define a near-neighbor dataset for downstream comparison (*n* = 185), rather than as a fixed cutoff for clade or transmission inference; for ST48/O113:H32, all available genomes meeting inclusion criteria at the time of retrieval were included (*n* = 11) (Table S1).

### 2.6. Statistical Analyses

Data were analyzed using GraphPad Prism version 9.5.0. One-way ANOVA was used to assess significant differences, with a *p*-value of <0.05 considered statistically significant.

## 3. Results

### 3.1. Clinical Characteristics and Treatment

**Figure 1:**
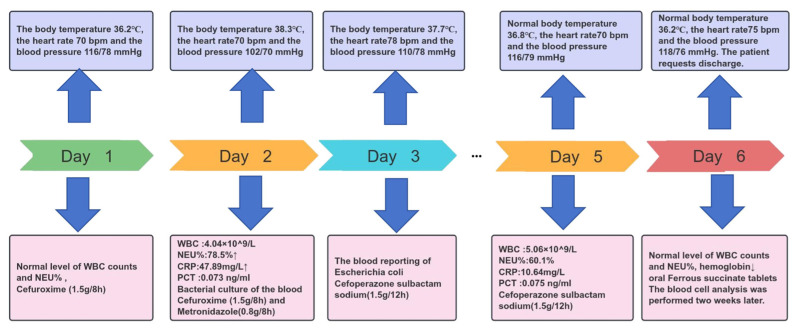
A timeline of the patient’s clinical presentation, evaluation, and treatment since cervical conization. Key results were as follows: (1) The patient had no fever on postoperative day 1 and received cefuroxime prophylactically. (2) On the second day, blood culture was collected after the patient developed a fever, and cefuroxime and metronidazole were combined for anti-infection. (3) On postoperative day 3, blood cultures yielded *E. coli*, and antibiotic therapy was adjusted accordingly. (4) On the fifth day, the patient’s body temperature was normal, and the blood test results were also normal. (5) On the sixth day, the patient was discharged.

The patient developed a fever (38.3 °C) on the second day after cervical conization. Laboratory tests revealed a white blood cell (WBC) count of 4.04 × 10^9^/L, with neutrophils accounting for 78.5%, lymphocytes 19.8%, and monocytes 1.7%. Inflammatory markers were elevated, with a C-reactive protein (CRP) level of 47.89 mg/L (reference range, <10 mg/L) and a procalcitonin (PCT) level of 0.073 ng/mL (reference range, <0.05 ng/mL). Based on these clinical symptoms and laboratory findings, empirical antibiotic therapy was initiated promptly (Figure 1). On postoperative day 3, *E. coli* was identified in the blood cultures, and antibiotic therapy was immediately adjusted to cefoperazone/sulbactam. Antibiotic susceptibility testing revealed that HBFY-1 was susceptible to cefoperazone/sulbactam, whereas HBFY-2 was resistant. Although HBFY-2 was non-susceptible to cefoperazone/sulbactam in vitro, the patient’s body temperature returned to normal on postoperative day 5 after three days of cefoperazone/sulbactam treatment. This temporal association is reported descriptively and should not be interpreted here as direct evidence of activity against HBFY-2.

### 3.2. Phenotypic and Genomic Characterization of the Isolates

**Figure 2:**
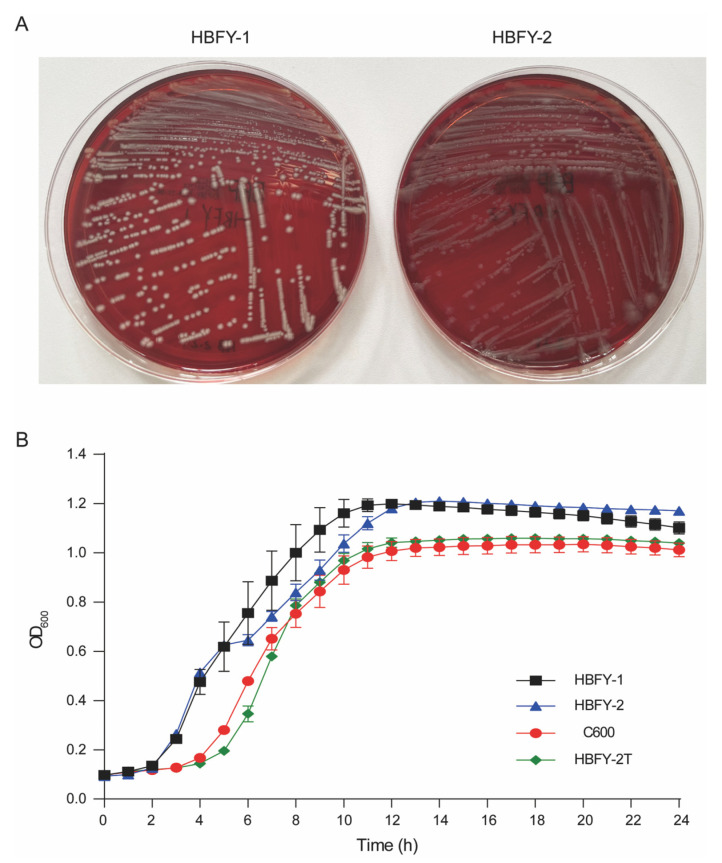
Colony morphology and growth curves of *E. coli* strains HBFY-1, HBFY-2, C600, and HBFY-2T (*bla*_NDM-5_-positive transconjugant derived from HBFY-2): (**A**) Colony morphology of HBFY-1 and HBFY-2 on blood agar plates. (**B**) Growth curves of *E. coli* strains HBFY-1, HBFY-2, C600, and HBFY-2T in LB broth over 24 h. Data are presented as mean ± standard deviation (SD).

The two bloodstream *E. coli* isolates exhibited distinct colony morphologies on blood agar: HBFY-1 formed white colonies, whereas HBFY-2 formed gray colonies (Figure 2A). Although selective media such as MacConkey agar were tested, the isolates could not be reliably distinguished. Growth kinetics did not differ significantly between HBFY-1 and HBFY-2 (*p* = 0.7715) (Figure 2B). Whole-genome comparison confirmed that both isolates belonged to *E. coli* at the species level (ANI 99.1%; dDDH 94.4%), whereas sequence typing and serotyping assigned them to distinct lineages (ST744/O101:H9 vs. ST48/O113:H32) (Table S2). In contrast, the reference strain *E. coli* DSM 30083 showed lower similarity (ANI 96.8% and 96.7%; dDDH 75.1% and 75.4% relative to HBFY-1 and HBFY-2, respectively) (Table 1).

HBFY-1 had a genome size of 4,843,550 bp assembled into 92 contigs, with a GC content of 50.81% and 4552 coding sequences (CDSs). Plasmid replicon typing identified IncFIB (AP001918) and IncFIC (FII), and no carbapenemase-encoding genes were detected. In contrast, HBFY-2 had a genome size of 4,818,223 bp assembled into 84 contigs, with a GC content of 50.69% and 4552 CDSs. It carried an IncI1/IncQ1/IncX1/IncY plasmid profile and notably harbored *bla*_NDM-5_. Phenotypic confirmatory testing showed that HBFY-1 was positive for ESBL production but negative for carbapenemase activity, whereas HBFY-2 was positive for metallo-carbapenemase activity but negative for ESBL production.

Antimicrobial susceptibility testing revealed that both isolates exhibited MDR phenotypes but with distinct profiles (Table 2). HBFY-2 was resistant to carbapenems (imipenem and meropenem) and most β-lactams, whereas HBFY-1 remained susceptible to carbapenems and β-lactam/β-lactamase inhibitor combinations but was resistant to fluoroquinolones. Both HBFY-1 and HBFY-2 remained susceptible to all tested aminoglycosides and showed intermediate susceptibility to colistin.

### 3.3. Phylogenomic Context of E. coli ST744/O101:H9 and ST48/O113:H32

**Figure 3:**
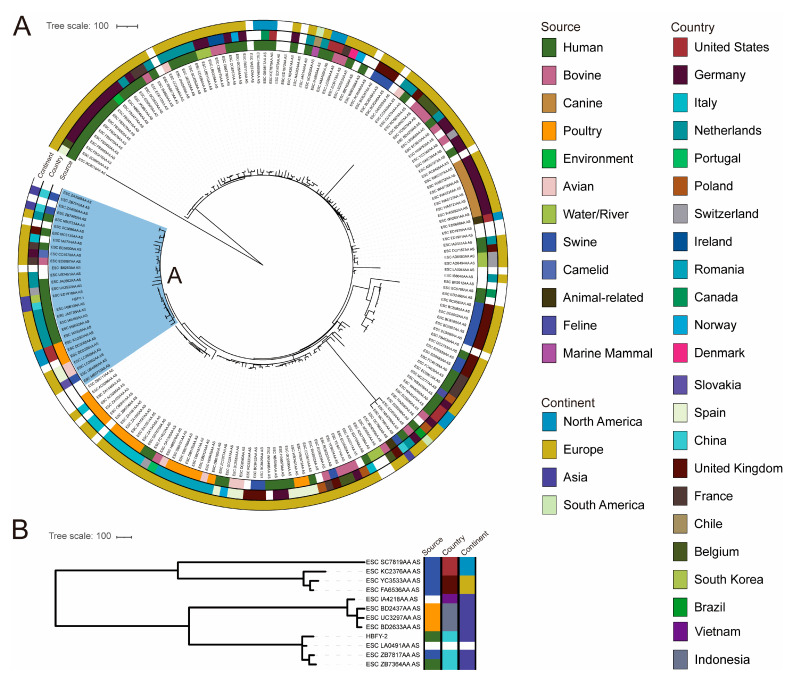
Core-genome SNP-based phylogenetic trees of *E. coli* ST744/O101:H9 and ST48/O113:H32: (**A**) Phylogeny of ST744/O101:H9 genomes. The clade containing HBFY-1 (Clade A; *n* = 29) is highlighted. (**B**) Phylogeny of ST48/O113:H32 genomes (*n* = 12). The outer rings represent source, country, and continent.

We constructed a recombination-filtered core-genome SNP phylogeny for global *E. coli* ST744/O101:H9 genomes, restricting the dataset to genomes within ≤60 core-genome SNPs of HBFY-1 (reference). In this analysis, HBFY-1 was placed within a monophyletic clade (Clade A, *n* = 29) (Figure 3A). Clade A included genomes from Europe (*n* = 17), Asia (*n* = 3), and North America (*n* = 2) and spanned multiple sources, including humans (*n* = 12), swine (*n* = 3), poultry/avian (*n* = 5), and sporadic bovine (*n* = 1) and camelid (*n* = 1) isolates. Within Clade A, HBFY-1 clustered most closely with the human-derived genome ESC_ED1916AA_AS (Asia/South Korea).

For ST48/O113:H32, a recombination-filtered core-genome SNP phylogeny was generated for 11 publicly available genomes using HBFY-2 as the reference. The resulting tree separated the ST48/O113:H32 genomes into two major clusters (Figure 3B). One cluster contained most isolates from Asia (*n* = 7), with one additional isolate lacking continent annotation. Within this Asia-predominant cluster, HBFY-2 grouped with two China-derived genomes, including one human isolate and one swine isolate.

### 3.4. Distinct Resistome Profiles of E. coli ST744/O101:H9 and ST48/O113:H32

Presence/absence-based clustering resolved two lineage-specific resistome groups (ST48 vs. ST744) (Figure S1). Within ST744, a highly conserved MDR “backbone” was observed: quinolone QRDR substitutions were fixed in all ST744 genomes (*gyrA*_S83L/*gyrA*_D87N/*parC*_S80I/*parC*_A56T, 186/186), and *sul2* (174/186) along with aminoglycoside resistance genes *aph(6)-Id* and *aph(3″)-Ib* were prevalent (175/186 and 174/186, respectively). HBFY-1 (ST744, human, China) clustered within the dominant ST744 MDR group and carried the canonical backbone but was distinguished by *tet*(A), *qnrS2*, and *bla*_CTX-M-27_. In contrast, ST48 (O113:H32) exhibited a lower and more heterogeneous AMR gene burden. Notably, *qacE*Δ1–*sul1* was detected in only two ST48 genomes, one of which was HBFY-2 (ST48, human, China). HBFY-2 was also the sole ST48 genome carrying *bla*_NDM-5_ (1/12), co-occurring with *ble* and a composite resistance module comprising *dfrA12*/*aadA2*/*sul3*/*cmlA1*, consistent with acquisition of a compact mobile MDR element (Table S2).

### 3.5. Architecture and Transferability of the bla_NDM-5_ Plasmid

**Figure 4:**
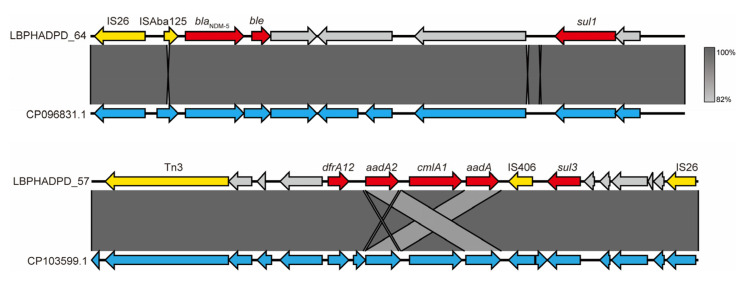
Comparative analysis of two resistance modules within plasmid bin AA378 from HBFY-2. LBPHADPD_64 aligned to *bla*_NDM-5_ plasmid CP096831.1, and LBPHADPD_57 aligned to *E. coli* plasmid CP103599.1. Resistance genes are shown in red, insertion sequences/transposition or recombination-related genes in yellow, and other proteins in gray; shaded regions indicate nucleotide identity between sequences.

To characterize the *bla*_NDM-5_-carrying plasmid in strain HBFY-2, a 121,475 bp plasmid bin AA378 consisting of six contigs was reconstructed (Table S3). Replicon typing identified a single IncY replicon on the 82,145 bp contig (LBPHADPD_23), which shared high sequence similarity with the IncY-like plasmid CP030183.1 (92% coverage; 98.13% identity) (Figure S2). Resistance gene annotation showed that the determinants were localized within two distinct modules. The first module contained *bla*_NDM-5_ together with *ble*, *sul1*, and *qacE*Δ1-*sul1*, flanked by insertion sequences including IS*26* and IS*Aba125*. This *bla*_NDM-5_-associated region was nearly identical to previously reported modules (e.g., CP096831.1; 100% coverage; 99.99% identity) (Figure 4). A second MDR module harboring *dfrA12*, *aadA2*, *cmlA1*, and *sul3* was identified. This locus exhibited near-complete identity to published *E. coli* plasmids (e.g., CP103599.1; 100% coverage; 99.99% identity), accompanied by multiple mobile genetic elements (MGEs) such as transposases and recombinases (Figure 4).

We then experimentally validated the transferability of the *bla*_NDM-5_–associated element identified in the reconstructed plasmid bin. Conjugation assays demonstrated successful transfer of *bla*_NDM-5_ from HBFY-2 into the recipient strain *E. coli* C600, and acquisition was confirmed by PCR. Additional conjugation assays showed that the *bla*_CTX-M-27_-associated element in HBFY-1 was also transferable to *E. coli* C600 under cefotaxime selection, with a conjugation frequency of 5.3 × 10^−2^ (Table 3). Phenotypic testing further confirmed that the transconjugant HBFY-2T was positive for carbapenemase production, consistent with the acquisition of the *bla*_NDM-5_-carrying element. The conjugation frequency was 4.6 × 10^−6^ (Table 3). Growth comparisons showed significant differences between HBFY-2 and both C600 and the transconjugant HBFY-2T (*p* < 0.0001), whereas C600 and HBFY-2T did not differ (*p* = 0.8297), indicating no detectable fitness cost associated with plasmid acquisition under the conditions tested (Figure 2B). Consistent with plasmid-mediated transfer, the transconjugant HBFY-2T retained resistance to β-lactams and carbapenems (including imipenem and meropenem), matching the *bla*_NDM-5_-linked phenotype of the donor strain. In contrast, susceptibility to piperacillin/tazobactam was restored, and susceptibility to aminoglycosides and fluoroquinolones shifted from intermediate to susceptible (Table 2). The recipient strain C600 remained susceptible to all tested antibiotics.

## 4. Discussion

Cervical conization is a standard intervention for cervical precancerous lesions; however, disruption of the cervical barrier and perioperative factors may increase susceptibility to postoperative infections, including BSIs [46,47]. *E. coli* is a common cause of BSIs, and carbapenem-resistant strains, particularly those producing NDM, may further complicate postoperative management [48,49]. In this single-case study, recovery of two *E. coli* isolates from blood cultures, including one *bla*_NDM-5_-positive isolate, suggests the potential value of combining routine diagnostics with timely resistance-mechanism characterization to define isolate-specific resistance features that might otherwise be overlooked.

Because this study was based on a single clinical case, the recovery of two *E. coli* isolates assigned to distinct lineages should be interpreted cautiously, and mixed bloodstream infection cannot be confirmed on the basis of the available evidence. A notable clinical observation in this case was the patient’s prompt defervescence after cefoperazone/sulbactam (CPZ/SAM) therapy, despite recovery of an *E. coli* isolate (HBFY-2) that was not susceptible to CPZ/SAM in vitro. This apparent clinical–microbiological discordance should be interpreted with caution [50]. The early clinical improvement may have been influenced by multiple factors, including host clearance, source control, and activity of CPZ/SAM against the other recovered isolate, HBFY-1, rather than reliable activity against the carbapenemase-producing isolate [51,52,53]. Therefore, the observed defervescence should not be considered evidence supporting CPZ/SAM as an appropriate targeted therapy for carbapenemase-producing *E. coli* [54,55]. Once carbapenemase production is confirmed and susceptibility results are available, treatment decisions should be guided by isolate-specific susceptibility data together with the overall clinical context.

Despite broadly comparable genome size and gene content, the two isolates belonged to ST744/O101:H9 and ST48/O113:H32, respectively, and showed distinct lineage-associated resistome patterns. In ST744, the near fixation of canonical QRDR substitutions across the dataset is consistent with sustained fluoroquinolone selection, a pattern commonly reported in settings with repeated antimicrobial exposure across human and animal sectors [56,57]. In contrast, ST48/O113:H32 showed a lower and more heterogeneous AMR gene burden, and class 1 integron-associated signatures (e.g., *qacE*Δ1-*sul1*) were uncommon in this small panel (*n* = 12), suggesting that extensive multidrug resistance is not uniformly represented in this lineage [58,59]. Notably, *bla*_NDM-5_ was detected only in HBFY-2 within ST48/O113:H32, suggesting that carbapenem resistance in this background may reflect a relatively recent and infrequent acquisition event in the available dataset rather than a lineage-wide feature.

In HBFY-2, *bla*_NDM-5_ was located on a conjugatively transferable platform and conferred the expected carbapenem-resistant phenotype in the recipient. The surrounding genetic context contained insertion sequence-associated features suggestive of IS-mediated mobilization of *bla*_NDM_-bearing regions, a mechanism widely involved in dissemination among *Enterobacterales*. The high similarity of this *bla*_NDM-5_ region to previously reported sequences is consistent with horizontal acquisition rather than local evolution in this ST48/O113:H32 background. Most previous reports identify IncX3 plasmids as the predominant vehicles for *bla*_NDM-5_, with IncF-type backbones also described in some settings [19,60]. However, recent studies suggest that *bla*_NDM-5_ genetic contexts are highly mobile and can occur across diverse plasmid replicon types, likely reflecting MGE-mediated dissemination shaped by local plasmid ecology [11,61,62]. In our isolate, *bla*_NDM-5_ was associated with an IncY-like plasmid scaffold, aligning with the view that this resistance determinant can be accommodated by non-canonical plasmid backgrounds and suggesting that surveillance could benefit from incorporating mobilizable resistance modules alongside traditional plasmid replicon typing.

Conjugation into C600 transferred only a subset of the donor resistance profile, suggesting that additional resistance determinants in HBFY-2 were not co-transferred and may be chromosomal or located on other plasmids. No detectable growth disadvantage was observed in the transconjugant under the conditions tested, which may indicate that the acquired element can be maintained with limited short-term fitness cost in this experimental setting. Together, these findings suggest that the *bla*_NDM-5_-carrying element may have the capacity to persist and disseminate. Additional studies under in vivo or other relevant conditions are needed to clarify its broader significance.

Finally, we considered both isolates in a broader phylogenomic context to better understand their circulation patterns and possible ecological links. For ST744/O101:H9, the ≤60 cgSNP near-neighbor dataset placed HBFY-1 within a monophyletic clade containing genomes from multiple continents and host sources. This observation is in line with previous genome-based studies showing that O101:H9, including ST744, has been recovered from animal-associated collections and that ST744/O101:H9 strains can show close genomic relatedness across household or cross-host settings [63,64]. For ST48/O113:H32, the phylogeny resolved two major clusters, with HBFY-2 grouping within an Asia-predominant cluster that included human and swine genomes from China. Although such clustering does not imply direct transmission, it is consistent with broader evidence that some *E. coli* lineages may circulate across human and livestock reservoirs, supporting the relevance of a One Health perspective when interpreting their distribution [65,66].

Our study has several limitations that should be considered when interpreting these findings. First, because this was a single-case investigation, the available evidence was insufficient to determine definitively whether the recovery of two *E. coli* lineages from blood cultures represented a true mixed infection. Second, short-read-based plasmid reconstruction remained fragmented, limiting the complete resolution of plasmid architecture and the genetic context of certain resistance modules. Third, our in vitro fitness assays were performed under defined laboratory conditions and may not fully reflect the stability or fitness effects of the transferred element in vivo or under other selective pressures. Accordingly, the findings of this study should be regarded as case-specific and hypothesis-generating, and broader generalization will require additional evidence from larger cohorts and more completely resolved genomic analyses.

## 5. Conclusions

In this single-case study, two genetically distinct *E. coli* isolates (ST744/O101:H9 and ST48/O113:H32) were recovered from blood cultures obtained during a postoperative febrile episode after cervical conization. The ST48 isolate carried a conjugatively transferable *bla*_NDM-5_-associated element located on an IncY-like plasmid scaffold, which conferred carbapenem resistance in the recipient and showed no detectable short-term fitness cost under the tested conditions. Because this study was based on a single clinical case, these findings should not be generalized beyond the available evidence and do not permit definitive confirmation of mixed bloodstream infection. Despite these limitations, this case highlights the potential utility of combining susceptibility testing with timely genomic characterization to identify mobilizable carbapenem-resistance platforms in routine clinical practice.

## Figures and Tables

**Table 1:** Average nucleotide identity (ANI) and digital DNA-DNA hybridization (dDDH) analysis of strains HBFY-1, HBFY-2, and *E. coli* DSM 30083 (%).

ANI/dDDH	HBFY-1	HBFY-2	*E. coli* DSM 30083
HBFY-1	100/100	99.1/94.4	96.8/75.1
HBFY-2	99.1/94.4	100/100	96.7/75.4
*E. coli* DSM 30083	96.8/75.1	96.7/75.4	100/100

**Table 2:** Minimal inhibitory concentrations (MICs) and antimicrobial susceptibility profiles of *E. coli* strains HBFY-1, HBFY-2, HBFY-2T (*bla*_NDM-5_-positive transconjugant), and *E. coli* C600.

Antimicrobial Class/Agent	MICs (µg/mL) [Antimicrobial Susceptibility]			
	HBFY-1	HBFY-2	HBFY-2T	*E. coli* C600
**Aminoglycosides**				
Amikacin	≤2 [S]	≤2 [S]	≤2 [S]	≤2 [S]
Gentamicin	≤1 [S]	≤1 [S]	≤1 [S]	≤1 [S]
Tobramycin	≤1 [S]	≤1 [S]	≤1 [S]	≤1 [S]
**Penicillins**				
Ampicillin	≥32 [R]	≥32 [R]	≥32 [R]	≤2 [S]
Piperacillin	≥128 [R]	32 [R]	16 [SDD]	≤4 [S]
**Monobactams**				
Aztreonam	16 [R]	≤1 [S]	≤1 [S]	≤1 [S]
**Cephalosporins**				
Cefazolin	≥64 [R]	≥64 [R]	≥64 [R]	2 [S]
Cefuroxime	≥64 [R]	≥64 [R]	≥64 [R]	4 [S]
Cefepime	2 [S]	16 [R]	16 [R]	≤1 [S]
Cefotetan	≤4 [S]	≥64 [R]	≥64 [R]	≤4 [S]
Ceftazidime	32 [R]	≥64 [R]	≥64 [R]	≤1 [S]
Ceftriaxone	≥64 [R]	≥64 [R]	≥64 [R]	≤1 [S]
Cefiderocol	0.125 [S]	0.06 [S]	0.06 [S]	0.06 [S]
**Carbapenems**				
Imipenem	≤1 [S]	≥16 [R]	≥16 [R]	≤1 [S]
Meropenem	≤0.25 [S]	≥16 [R]	≥16 [R]	≤0.25 [S]
**β-lactam/β-lactamase inhibitors**				
Ceftazidime/Avibactam	0.125/4 [S]	≥256/4 [R]	≥256/4 [R]	0.125/4 [S]
Imipenem/Relebactam	0.125/4 [S]	≥32/4 [R]	≥32/4 [R]	0.06/4 [S]
Ampicillin/Sulbactam	8/4 [S]	≥32/16 [R]	≥32/16 [R]	≤2/1 [S]
Ticarcillin/Clavulanic acid	16/2 [S]	≥128/2 [R]	≥128/2 [R]	≤8/2 [S]
Piperacillin/Tazobactam	≤4/4 [S]	64/4 [R]	8/4 [S]	≤4/4 [S]
Cefoperazone/Sulbactam	≤8/4 [S]	≥64/32 [R]	≥64/32 [R]	≤8/4 [S]
**Fluoroquinolones**				
Ciprofloxacin	≥4 [R]	0.5 [I]	≤0.25 [S]	≤0.25 [S]
Levofloxacin	≥8 [R]	1 [I]	≤0.25 [S]	≤0.25 [S]
**Polymyxins**				
Colistin	2 [I]	2 [I]	1 [I]	1 [I]

Note: R, resistant; I, intermediate; S, susceptible; SDD, susceptible dose-dependent.

**Table 3:** Conjugation transfer frequencies of HBFY-1 and HBFY-2 using *E. coli* C600 as the recipient strain.

Donor	Recipient	Number of Bacterial Colonies (Dilution Factor)			CF
		MacConkey (SM)	MacConkey (CTX + SM)	MacConkey (MEM + SM)	
HBFY-1	C600	181 (x = 10^−5^)	96 (y = 10^−4^)	-	5.3 × 10^−2^
HBFY-2	C600	173 (x = 10^−5^)	-	80 (y = 1)	4.6 × 10^−6^

Note: SM, streptomycin; MEM, meropenem. CF, conjugation frequency; CF = (number of transconjugant bacterial colonies × 10x)/(number of recipient bacterial colonies × 10y), where x and y represent the dilution factors.

## Data Availability

The complete genome sequences of strain *E*. *coli* isolates (HBFY-1 and HBFY-2) sequenced in this study have been deposited in GenBank under the BioProject ID PRJNA1259136. Information for the publicly available genomes included in the comparative analysis is provided in Table S1.

## Supplementary Materials

The following supporting information can be downloaded at: https://www.mdpi.com/article/10.3390/pathogens15050476/s1, Figure S1: Distribution of antimicrobial resistance genes in *Escherichia coli* ST744/O101:H9 and ST48/O113:H32; Figure S2: IncY-like plasmid backbone of plasmid bin AA378; Table S1: Public *Escherichia coli* genomes used in this study; Table S2: Genetic characteristics of *Escherichia coli* HBFY-1 and HBFY-2; Table S3: Contig-level composition of the *bla*_NDM-5_-associated plasmid bin in *Escherichia coli* HBFY-2.
