# Supplementary material for: Two Multidrug-Resistant Escherichia coli Isolates from Blood Cultures After Cervical Conization in a Patient with Cervical Intraepithelial Neoplasia Grade II: Phenotypic and Genomic Characterization
Source: Pathogens. 2026 Apr 28;15(5):476. doi: 10.3390/pathogens15050476 (PMC13209812; doi:10.3390/pathogens15050476)
Supplement: Supplementary file 1 [file pathogens-15-00476-s001.zip › Additional file 2.pdf]

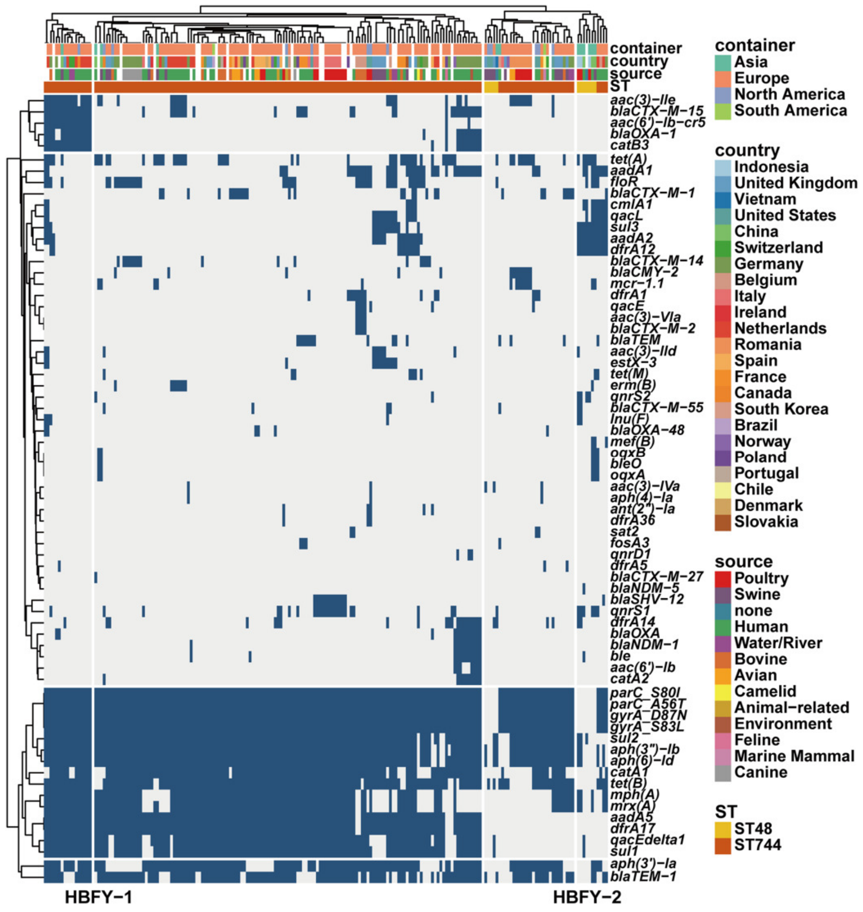

Figure S1. Distribution of antimicrobial resistance genes in *Escherichia coli* ST744/O101:H9 and ST48/O113:H32.

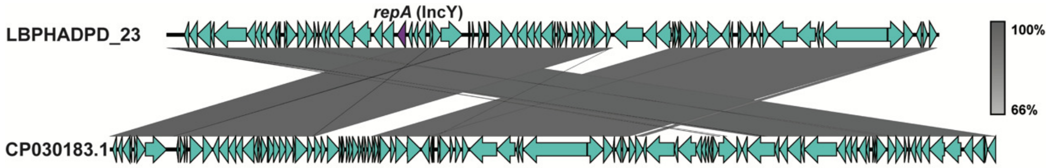

Figure S2. IncY-like plasmid backbone of plasmid bin AA378. The IncY-bearing contig LBPHADPD_23 (82,145 bp) was aligned to the complete IncY-like plasmid CP030183. 1. repA (IncY) is shown in purple.
